# Validation of portable, semi-dry electrode-based electroencephalography device for its application in brain–computer interface solutions

**DOI:** 10.1038/s41598-026-52672-8

**Published:** 2026-07-24

**Authors:** János Rokai, Melinda Rácz, Melinda Becske, János Csipor, Csaba Márton Köllőd, István Ulbert, Gergely Márton

**Affiliations:** 1 Institute of Cognitive Neuroscience and Psychology, HUN-REN Research Centre for Natural Sciences, Magyar Tudósok krt. 2, Budapest, 1117 Hungary; 2 MindRove Kft., Hédervári út 43, Gyor, 9026 Hungary; 3 School of PhD Studies, Semmelweis University, Üllői út 26, Budapest, 1085 Hungary; 4 Selye János Doctoral College for Advanced Studies, Semmelweis University, Üllői út 22, Budapest, 1085 Hungary; 5 Department of Psychiatry and Psychotherapy, Semmelweis University, Balassa u. 6, Budapest, 1083 Hungary; 6 Nyiro Gyula National Institute of Psychiatry and Addictology, Neurocognitive Research Centre, 59-61 Lehel Utca, Budapest, 1135 Hungary; 7 Faculty of Information Technology and Bionics, Pázmány Péter Catholic University, Práter Utca 50/a, Budapest, 1083 Hungary; 8 Department of Neurosurgery and Neurointervention, Faculty of Medicine, Semmelweis University, Amerikai út 57, Budapest, 1145 Hungary

**Keywords:** Electroencephalography, Portable electroencephalography device, Visually evoked potential, Transient visually evoked potential, Event-related potential, P300, Motor execution, Brain–computer interface, Biological techniques, Engineering, Neuroscience

## Abstract

In recent years, commercial lightweight electroencephalography (EEG) headsets are gaining popularity in neuroscience. These devices commonly utilize only a few dry electrodes in specific locations and signal quality is often inferior compared to that of their traditional counterparts. In this study, we wanted to assess the feasibility of portable, paste-less, passive electrode-based EEG headset MindRove vision (VSN) for laboratory use. Three paradigms were implemented for acquiring visual evoked potential (VEP), P300 event-related potential and motor execution task (ME) related cortical patterns. Measurements were taken by using VSN, with wet-electrode system mBrainTrain SMARTING applied as reference. The performance of the devices was assessed by using signal-to-noise ratio (SNR) for VEP and P300 while support vector machine, random forest and convolutional neural network-based classifiers were fit to ME data. The SNRdB (i.e. SNR expressed in decibels) of VSN was greater for both VEP and P300, by a margin of 1.998 and 2.845 dB, respectively. There was a significant difference between VEP signal amplitude levels and SNRdB, P300 SNR and SNRdB in favor of VSN. Average accuracy of the sorters were 78.8% for VSN and 80.9% for SMARTING; the difference was not significant. The application of VSN is feasible for use in research besides qualitative exploration.

## Introduction

Brain–computer interfaces (BCIs) are assistive devices that aim to improve the quality of life of people living with disabilities affecting motor control^1^, such as tetraplegia^2–4^ or amyotrophic lateral sclerosis^5^. These systems intend to bypass the natural neuromuscular pathways, enabling the control of a computer program or an actuator (e.g. a robotic arm^3,4^ or exoskeleton^2^) using neural signals corresponding to some control command. A large body of BCIs use electroencephalography (EEG) as signal acquisition modality due to its non-invasiveness^6^. Since EEG measures the aggregated activity of neurons in a few-centimeter radius^7^, that gets filtered by the meninges, skull and scalp^6^, implemented controls cannot be as intricate as solutions using signals captured by either electrocorticography or intracranial electrodes^8^. On the other hand, there are a handful of widely applied EEG-BCI paradigms that exploit well-defined cortical patterns that can suffice for many users, such as steady-state visually evoked potentials, event-related potentials and motor imagery^9^.

Further advantages of EEG-BCIs are that they utilize relatively low-cost signal acquisition devices that can be installed and removed any time, without any risk^8^. On the other hand, traditional EEG caps contain tens of (most commonly, 32 or 64) electrodes that can be brought into contact with the scalp by using electrolyte gel that can be lengthy and cumbersome to apply. As a solution for this problem, in the recent years, EEG systems utilizing dry electrodes have appeared on the market, offering also increased portability at a lower price. As a downside, these devices typically have a more limited set of channels and signal quality inferior to traditional EEG systems. Despite of that, these solutions slowly find their way into BCI research.

The most researched portable EEG headset is definitely the Emotiv EPOC (and its variants such as EPOC + or Flex)^10^, that uses 14 saline-based electrodes attached to the frame with plastic arms. Signals recorded with this device has been validated/examined for use in relation to various paradigms, such as visual^11–14^ and auditory^15–18^ oddball (P300), N400^19^, N170^20^, SSVEP^18^ and motor imagery^18,21^ paradigms. Additionally, the appliance was tested for evaluating drowsiness^22^ and musical pleasure^23^. In some cases, the original equipment was modified to adhere to the electrode configuration required by the task in question. EPOC has also been proved to provide excellent comfort, practicability and a compelling appearance^24,25^; on the other hand, it has been criticized for the lack of electrodes on the central areas and the rigidity of the plastic electrode holders that are unable to accommodate the head shape of various users^25,26^. Emotiv EPOC Flex has also been validated for various paradigms^27^, with its repeatability of measurement also assessed^28^. Another popular portable device, NeuroSky MindWave records EEG on a single channel above the prefrontal area, using a dry electrode. Nevertheless, it has been also validated for BCI research^29–31^, not infrequently in comparison to Emotiv EPOC, regarding signal quality^32^, cognitive load detecting capabilities^33^ and ergonomics^34^. InteraXon Muse, an ergonomic headband designed for use in mental health applications, has also been tested for use in tandem with visual oddball tasks^35,36^ and other paradigms^29,37^. Other commercial dry-electrode devices used in validation studies include the Cognionics Quick-20 system^38,39^, and PSBD Headband and Headphones^37^.

This paper aims at the validation of a lightweight, flexible, semi-dry, passive electrode-based EEG headband called vision (VSN), produced by the Hungarian biosensor manufacturer MindRove, in comparison with wet electrode-based headset mBrainTrain SMARTING (mBT). We intended to assess the capabilities of the device to capture signals usable for brain–computer interfacing tasks. Thus, we implemented three paradigms, namely, transient visually evoked potentials (VEP), a visual oddball paradigm for capturing event-related potential P300, and a motor execution (ME) paradigm involving the movement of both hands. Signal quality was benchmarked by using relevant forms of signal-to-noise ratio (SNR) for VEP and P300 data, whereas ME data, three machine learning classifiers (support vector machines—SVM, random forest—RF and convolutional neural networks—CNN) were fit and their overall accuracy was evaluated.

## Results

### VEP measurements

**Fig. 1:**
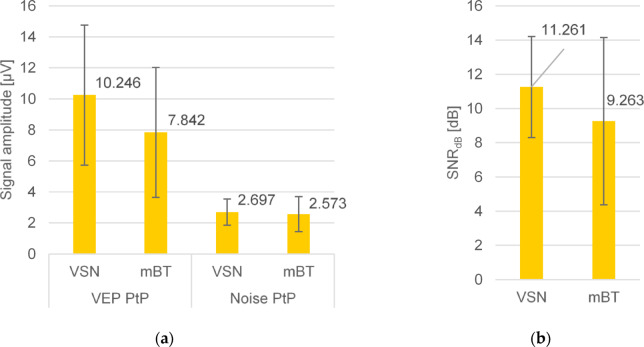
(**a**) Average signal (VEP) and noise peak-to-peak values and standard deviations and (**b**) average SNR_dB_ values and standard deviations for the two devices (VSN: MindRove vision, mBT: mBrainTrain SMARTING) across subjects.

Averages for signal and noise peak-to-peak values and signal-to-noise ratio given the two devices are shown in Fig. 1.

**Fig. 2:**
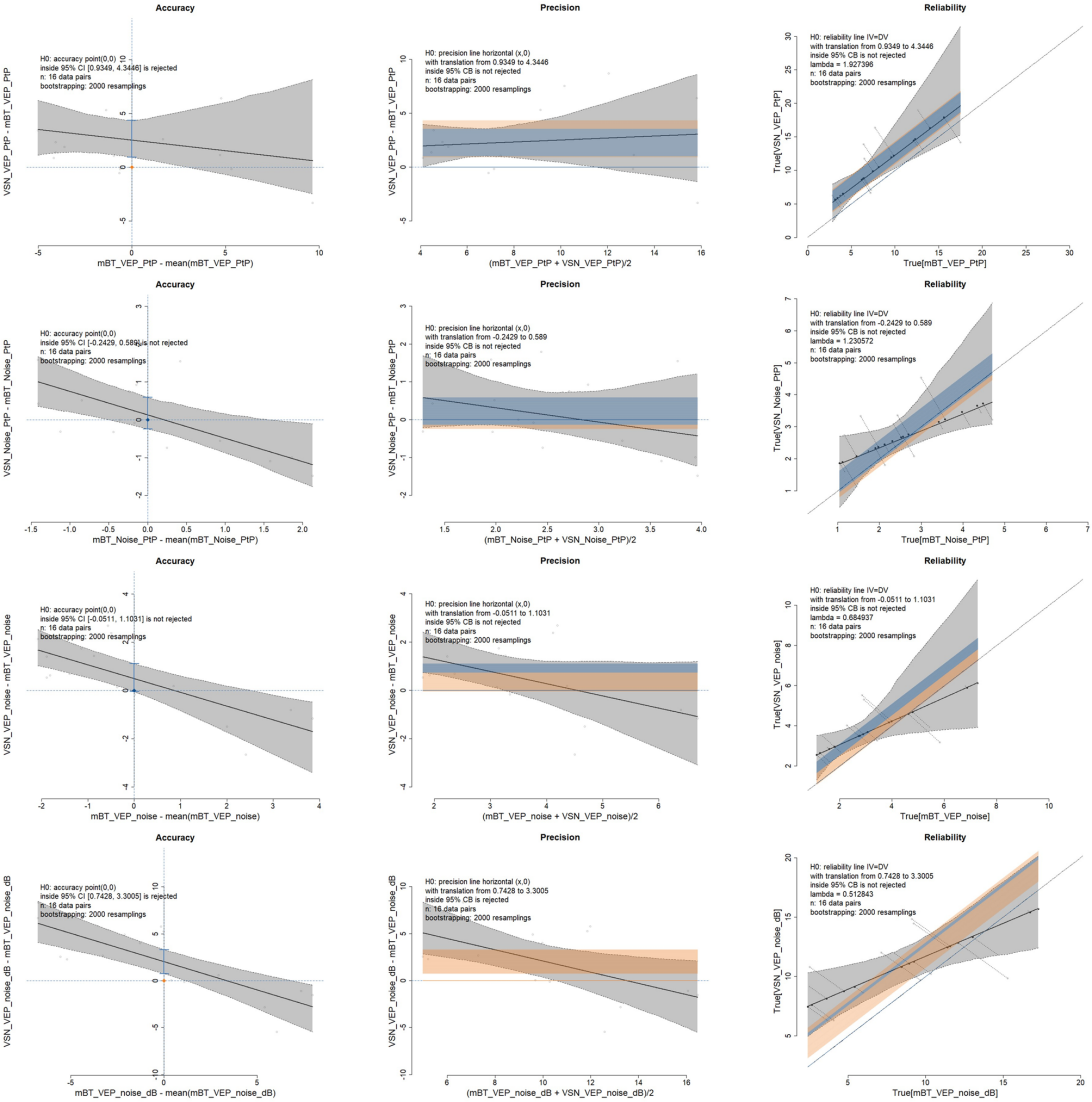
Accuracy, precision and bisector line agreement plots for the measured VEP variables. Rows, from top to bottom: difference of peak-to-peak VEP values; difference of peak-to-peak noise values; difference of signal-to-noise ratios; difference of signal-to-noise ratios converted to decibels for VSN and mBT.

We measured significantly greater VEP peak-to-peak values using VSN (10.246 µV) than mBT (7.842 µV) by a 2.404 µV margin. Based on the accuracy test (refer to the top left subplot of Fig. 2), we could observe a structural bias (with a (0, 0) point outside of the 95% confidence interval), thus the null hypothesis could be rejected. Precision and bisector line agreement tests (see the middle and right subplots of Fig. 2), however, showed that the variability of the measurement errors does not differ significantly for the two devices and that they are capable of measuring the same values in the same subjects, thus the two devices are interchangeable, given that the bias gets corrected.

Noise levels were nearly identical (2.697 and 2.573 µV), with mBT performing better by a 0.124 µV margin. Neither of the statistical methods showed significant differences between average values (see the second row of Fig. 2).

SNR was considerably greater for VSN, resulting in a 1.998 dB gap between the performance of VSN (11.261 dB) and mBT (9.263 dB). Interestingly, significant differences in accuracy and precision were observed in SNRdB values and not in simple SNR (see the third and fourth row of Fig. 2 for details).

### P300 measurements

**Fig. 3:**
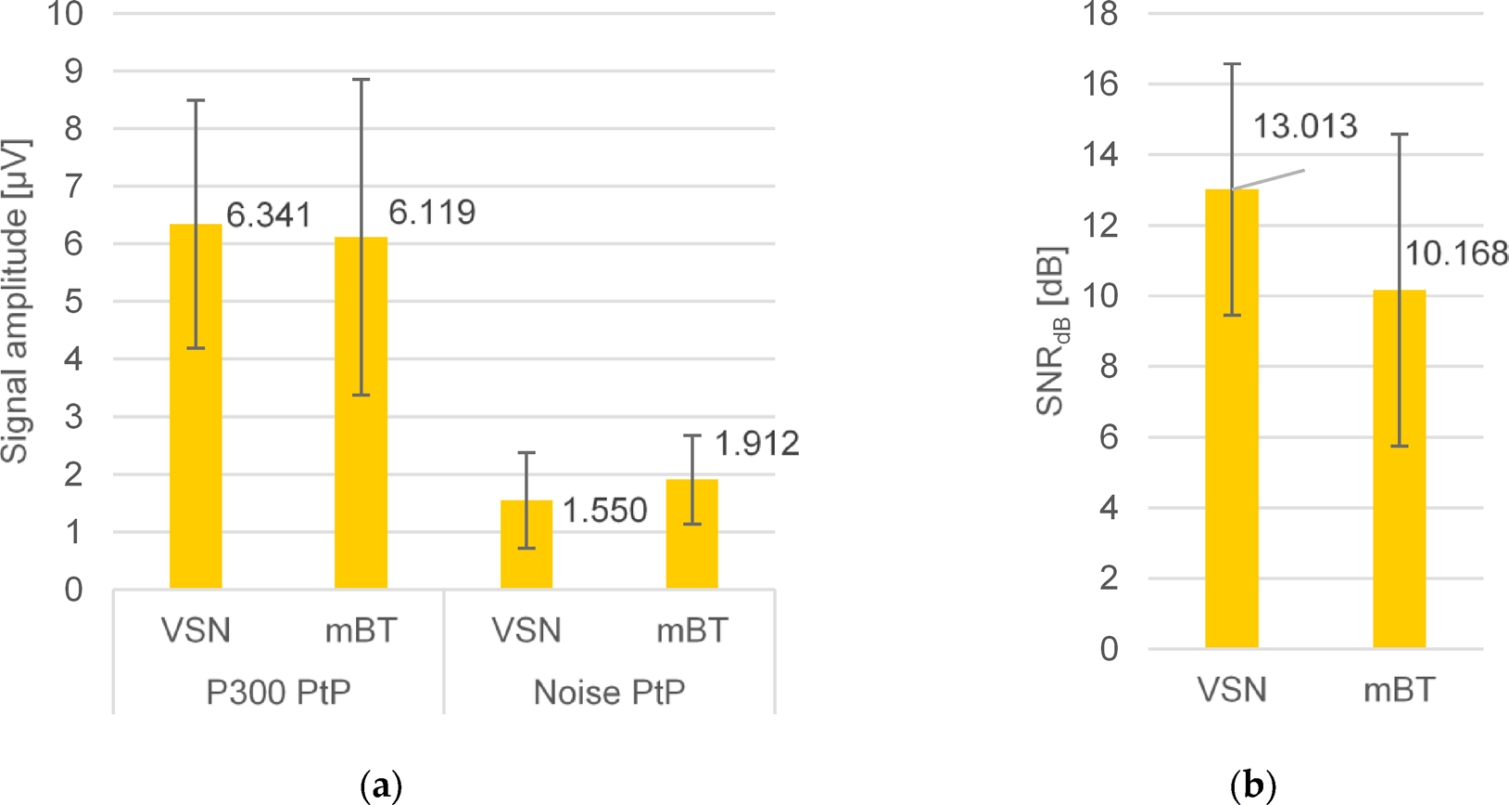
(**a**) Average signal (P300) and noise peak-to-peak values and standard deviations and (**b**) average SNR_dB_ values and standard deviations for the two devices (VSN: MindRove vision, mBT: mBrainTrain SMARTING) across subjects.

Averages for signal and noise peak-to-peak values and signal-to-noise ratio given the two devices are shown in Fig. 3.

**Fig. 4:**
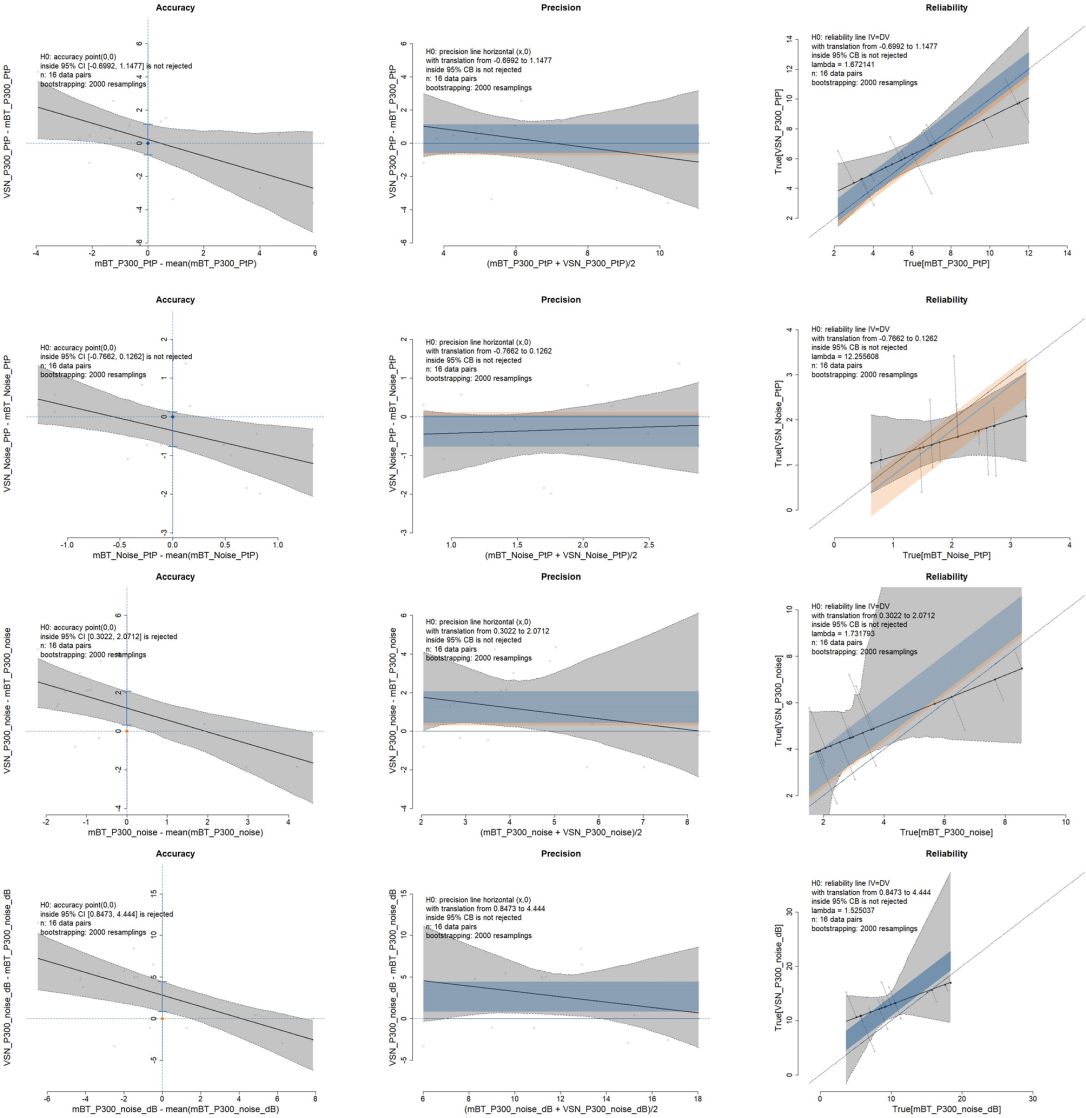
Accuracy, precision and bisector line agreement plots for the measured P300 variables. Rows, from top to bottom: difference of peak-to-peak P300 values; difference of peak-to-peak noise values; difference of signal-to-noise ratios; difference of signal-to-noise ratios converted to decibels for VSN and mBT.

P300 amplitudes were nearly identical (6.341 µV for VSN and 6.119 µV for mBT), with VSN performing better by a 0.222 µV margin; no significant difference was indicated by accuracy, precision and bisector line agreement tests (see the top row of Fig. 4). Although we measured slightly greater noise levels by using mBT (1.912 µV) than VSN (1.550 µV), by a 0.362 µV margin, this difference was not significant, either (see the second row of Fig. 4).

SNR was significantly better for VSN, resulting in a 2.845 dB gap between the performance of VSN (13.013 dB) and mBT (10.168 dB), confirmed by accuracy tests for both SNR and SNRdB. Precision and bisector line agreement tests (see the middle and right subplots of Fig. 4), however, showed that the variability of the measurement errors does not differ significantly for the two devices and that they are capable of measuring the same values in the same subjects, thus the two devices are interchangeable, given that the bias gets corrected. For details, see the third and fourth row of Fig. 4.

### ME measurements

**Fig. 5:**
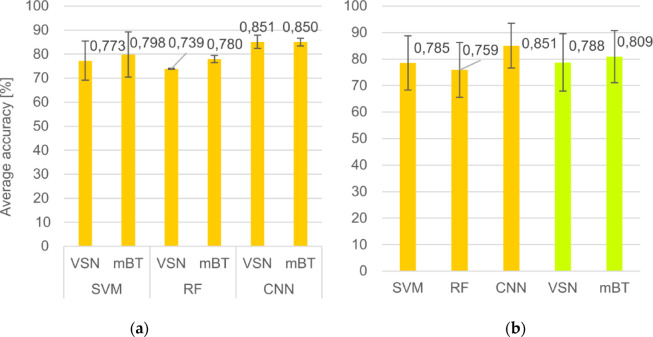
(**a**) Average accuracies and standard deviations for each classifier (SVM: support vector machines, RF: random forest, CNN: convolutional neural network) and device (VSN: MindRove vision, mBT: mBrainTrain SMARTING) across subjects; (**b**) average accuracies and standard deviation across sorter types (yellow columns) and devices (green columns).

Regarding ME measurements, there were greater differences in the average performance of sorters (SVM: 0.785, RF: 0.759, CNN: 0.851) then devices (VSN: 0.788, mBT: 0.809). The overall accuracy of mBT was greater by a 2.1% margin. Of the three sorters, CNNs were the most accurate, exceeding the performance of SVMs and RFs by 6.6% and 9.2%, respectively. There were a 2.7% difference between the average accuracy of SVMs and RFs. Details are shown in Fig. 5.

The LMM assuming the same variance for all subjects found a significant difference (*p* = 0.003) between the two devices, along with the algorithm type (*p* < 0.0001) For details, see Table 1. Contrary to this, based on results of the LMM that assumed different variance for the individual subjects, we found that the device type did not have a significant effect on average accuracy (*p *= 0.3982). The effect of algorithm type, however, reached significance level (*p* < 0.0001). For details, see Table 2.

**Table 1:** ANOVA results for the additive linear mixed model assuming the same variance for subjects.

	F-value	*p* value
Intercept	2020.6483	< 0.0001
Algorithm	55.8917	< 0.0001
Device	8.9214	0.003

**Table 2:** ANOVA results for the additive linear mixed model assuming different variance for subjects.

	F-value	*p* value
Intercept	2019.8512	< 0.0001
Algorithm	58.1109	< 0.0001
Device	0.7151	0.3982

The quality of the two models were compared by using the likelihood ratio test and we found that the model assuming different variance performed significantly better (*p* < 0.0001). For details, refer to Table 3.

**Table 3:** ANOVA results for the test between the additive linear mixed models assuming the same variance (top row) and different variance (bottom row) for subjects.

Model	df	AIC	BIC	LogLik	L.ratio	*p* value
Same	6	− 976.8996	− 951.9071	494.4498	57.44064	< 0.0001
Different	21	− 1004.3402	− 916.8665	523.1701		

Analogously to the latter additive model, the interaction LMM showed significance for algorithm type (*p* < 0.0001) but not for either device type (*p* = 0.2184) or the interaction to the former two (*p* = 0.0242). See Table 4 for further details. Upon comparing this model to the additive one, we found that the latter had better performance, although the difference did not reach the level of significance (*p* = 0.0585). For further details, see Table 5.

**Table 4:** ANOVA results for the linear mixed model assuming interaction between fixed effects different variance for subjects.

	F-value	*p* value
Intercept	2019.9478	< 0.0001
Algorithm	59.7463	< 0.0001
Device	1.5187	0.2184
Algorithm/Device	3.7537	0.0242

**Table 5:** ANOVA results for the test between the additive linear mixed model and another LMM assuming interaction between device and algorithm type. Both models assumed different variance for subjects.

Model	df	AIC	BIC	LogLik	L.ratio	*p* value
Additive	21	− 1034.967	− 947.3175	538.4835	5.676758	0.0585
Interaction	23	− 1036.644	− 940.6467	541.3219		

### ERP morphology and trial rejection

**Fig. 6:**
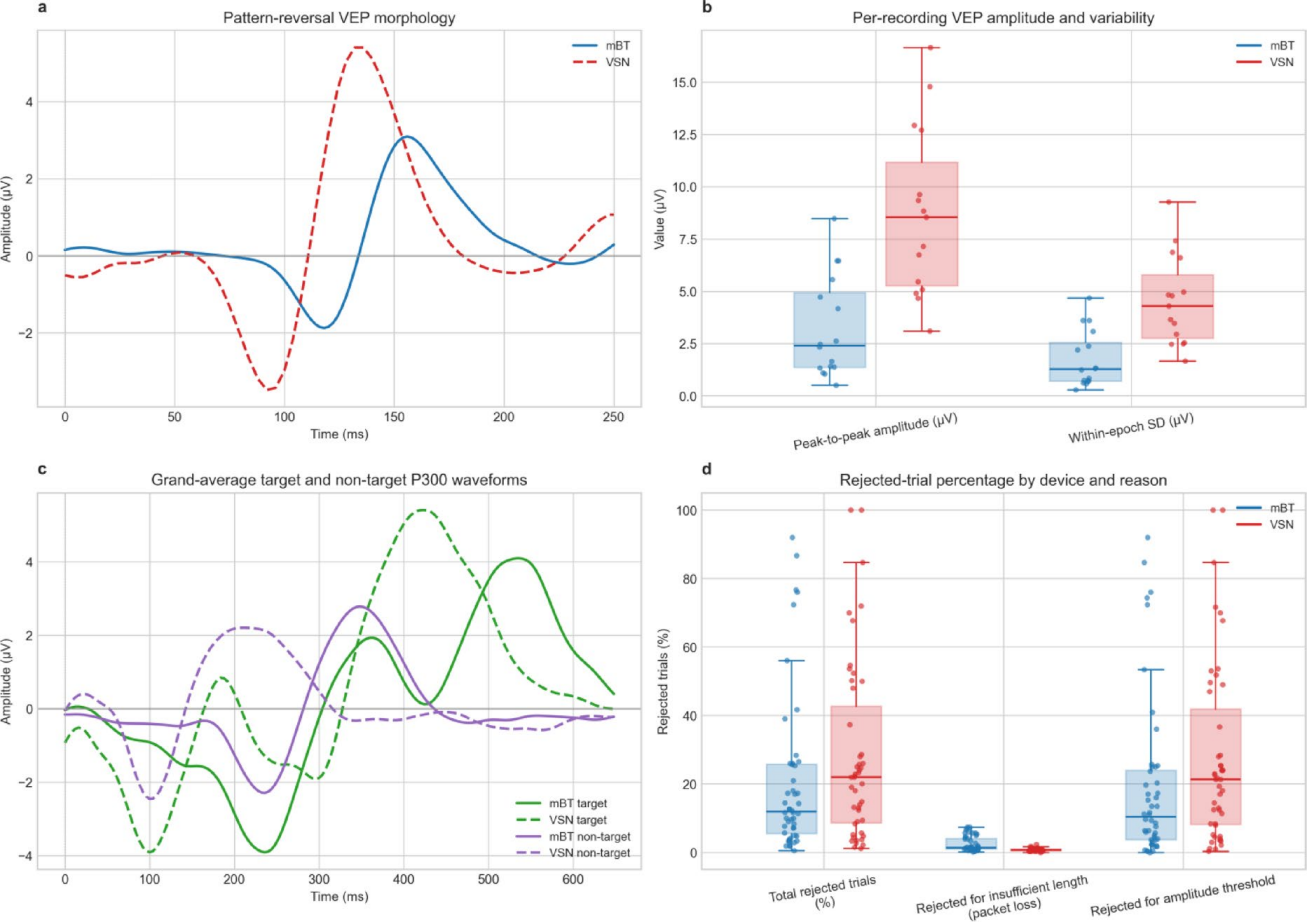
ERP morphology and trial-rejection summary for the two EEG systems. (**a**) Grand-average pattern-reversal VEP waveforms for mBT and VSN, computed from occipital channels (O1/O2). (**b**) Per-recording VEP amplitude and variability metrics, including peak-to-peak amplitude and within-epoch standard deviation. (**c**) Grand-average target and non-target P300 waveforms for mBT and VSN, averaged across channels C3, Cz, and C4. (**d**) Percentage of rejected trials for each device, shown overall and separately for rejection due to insufficient length (reflecting packet loss) and amplitude threshold violations. Points represent individual recordings; boxes indicate distribution across recordings.

To complement the SNR-based comparison, we examined the morphology of the averaged pattern-reversal VEP and visual-oddball P300 responses obtained with the two devices (Fig. 6). The grand-average VEP waveforms showed comparable overall morphology for both systems, with recognizable deflections in the expected latency range, while VSN exhibited larger peak-to-peak amplitudes. Similarly, the P300 paradigm yielded a clear separation between target and non-target responses for both devices, with a pronounced late positive component in the target condition.

We further quantified trial rejection to assess the impact of data loss and artifacts. Rejected trials were categorized based on insufficient length (reflecting packet loss) and amplitude threshold violations. While overall rejection rates remained moderate, packet-loss-related trial rejection may reduce the number of usable epochs and could affect timing-sensitive real-time BCI applications.

## Discussion

The aim of this research was to compare semi-dry, passive electrode-based lightweight EEG headband MindRove vision (VSN) to gel-based, passive system mBrainTrain SMARTING across three classic paradigms. For VEP measurements, we observed significant differences in accuracy, corresponding to greater signal peak-to-peak values for data recorded with VSN. Differences in precision and capability of the two devices to measure the same values did not reach significance, signifying interchangeability between the two instruments. There were also insignificant differences in measured noise and SNR values (with the latter closer to reach significance level). On the other hand, both accuracy and precision for SNRdB significantly differed. VEP amplitude values were also comparable to the range reported in literature. Odom^60^ specifies an approximately 20 µV peak-to-peak value while our measured variables were 10.246 µV for VSN and 7.842 for mBT, on average.

Regarding P300 measurements, the difference between signal and noise levels with respect to the measurement devices did not reach significance. Only SNR and SNRdB accuracy differed significantly for the two instruments, but precision and bisector line agreement tests confirmed the equivalence of the two devices. The relation of the measured amplitude to textbook peak-to-peak values was similar to that of the VEP. For example, Picton^69^ shows values up to approximately 15 µV in contrast to our 6.341 and 6.119 µV measured with VSN and mBT, respectively.

Machine learning classifiers yielded average accuracy values between 73.9 and 85.1% with CNNs exhibiting the best and RF the worst performance. The difference between the accuracy of the various classifiers proved to be significant, whereas the type of the signal acquisition instrument did not influence classification performance significantly.

Apart from assessing signal quality, we also have to discuss some practical and ergonomical considerations. Firstly, as it could be expected, VSN was easier and quicker to install. Although preparation time was not formally quantified, we consistently observed shorter setup times for the VSN system (approximately 10 min) compared to the mBT system (approximately 40 min) under our experimental conditions.The cause of this phenomenon might be that electrolyte gels require some time to penetrate into the scalp and provide good electrical contact. Moreover, since the reference and driven right leg electrodes of VSN and mBT do not match, signal recorded with mBT had to be re-referenced; in order to make this possible, we set two extra electrodes (TP9 and TP10) compared to VSN (11 electrodes instead of only 9). On the other hand, once electrical contact between gel-based electrodes and the skin is established, it cannot be disrupted easily. A drawback of VSN was that water was prone to evaporation and contacts had to be checked regularly, between measurements, and adjusted, if necessary, taking 1–3 extra minutes accordingly to the subject’s hair length or skin quality. Regarding rejected trials, the relatively high rejection rate was mainly driven by amplitude-threshold violations, which were more frequent for the semi-dry VSN.

Another characteristic property of VSN is that it applies electrodes that have to be firmly pressed against the scalp in order to keep impedances low, similarly to comb electrodes. This may introduce discomfort or even a mild headache during long-term use. This discomfort is not present in gel-based EEG systems. Additionally, frontal electrodes and the photoplethysmography module of the VSN left imprints in the forehead of the participants that required a few ten minutes to disappear entirely.

There are also a number of limitations we cannot leave unaddressed. Firstly, measurements were taking place separately due to the physical incompatibility of the two systems. Although there are examples of device modifications^17,19,20^, where holes were cut into a standard EEG cap where the electrode of EPOC contact to the scalp, we could not follow such a procedure. This is partly due to the closeness of the relevant electrodes of the two headsets and the size of VSN electrodes, i.e. cutting holes of sufficient size into the EEG cap would have a detrimental effect on its usability. Therefore, since there are research featuring data acquired from separate subject pools for the different devices^21,35^, we decided that recordings will be taken separately with the two devices, with mBT at last. This fixed order may also introduce biases into our measurements in the form of insufficient competence and/or mild anxiety at the first and boredom and/or fatigue at the second session. Additionally, we could not guarantee the identical placement of electrode for the two systems.

Secondly, the small size of the subject pool limits the credibility of our results. On the other hand, other research articles also feature a similar number of participants. Out of 24 publications about the validation of some portable headset (cited in the Introduction section), 10 featured a subject pool counting more than 16 participants. The balance of female and male participants (the fraction of the element number of the two genders, with the smaller number in the numerator) was also in the better half of this selection, with only 7 papers featuring a more balanced set of subjects. For details, see Table 6.

**Table 6:** Subject pool element number and gender balance comparison for literature on the validation of portable EEG devices.

Paper	Number of subjects	Gender balance
*Our research*	*16*	*0.778*
^11^	9	0.125
^12^	60	0.277
^13^	12	0.714
^14^	7	0.000
^15^	15	0.875
^16^	21	0.909
^17^	21	0.750
^18^	20	0.111
^19^	15	0.364
^20^	13	0.625
^21^	6	N.A
^23^	16	0.600
^27^	20	0.176
^28^	30	1.000
^29^	5	0.667
^30^	21	0.909
^31^	38	0.810
^32^	10	0.111
^33^	10	0.111
^35^	120	0.765
^36^	1000	0.919
^37^	11	0.833
^37^	13	0.300
^38^	12	0.714

Another important restriction of our research was the homogeneity of the subject pool in terms of demography, representing mainly young people from academia, being enrolled into tertiary or postgraduate education. That was the consequence of convenience sampling, however, it had to be considered.

## Materials and methods

### Data acquisition instruments

#### MindRove vision

**Fig. 7:**
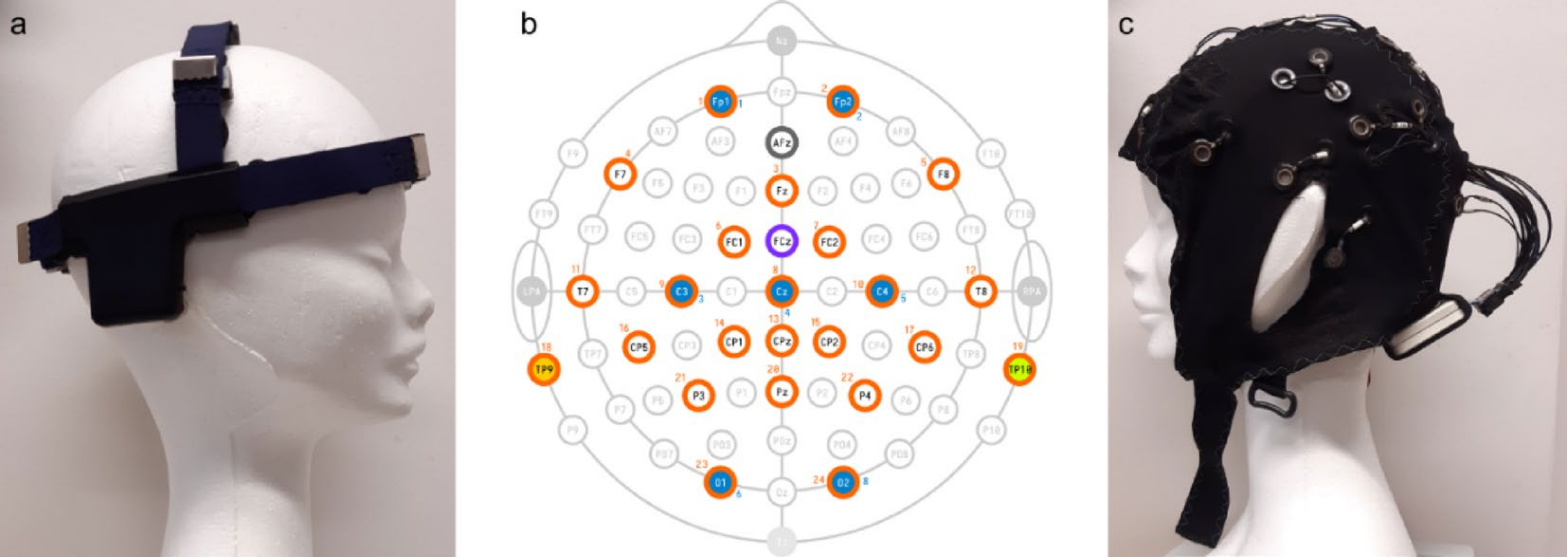
(**a**) Photograph of the MindRove vision; (**b**) combined montage of the MindRove vision and the mBrainTrain SMARTING. Sites filled with blue, yellow and green are the signal, reference and driven right leg electrodes of the MindRove vision, respectively. Similarly, sites outlined with orange, violet and dark grey are the signal, reference and DRL electrodes of the mBrainTrain SMARTING; (**c**) photograph of the mBrainTrain SMARTING.

MindRove vision (VSN, MindRove Kft., Győr, Hungary^40^) is a lightweight EEG headband electronically similar to the neuroMoon neurofeedback EEG headset^41^, MindRove arc^42,43^, and armband^44,45^. The photograph of the device is shown in Fig. 7a.

The headset includes seven electrodes positioned at Fp1, Fp2, C3, Cz, C4, O1 and O2 according to the 10–20 system. Reference and driven right leg electrodes are placed behind the left and right ears (TP9 and TP10), respectively. Device montage is shown in Fig. 1b. The electrodes are based on conductive fabric and platinum-iridium wires. They are strongly recommended to be wetted by using saline or water. The device acquires data with a resolution of 24 bits at a sampling rate of 250 Hz; the least significant bit (LSB) equals 0.045 µV. Contact impedances are measured at 31.2 Hz.

Besides the EEG measurement capabilities assessed in this paper, the device is equipped with an inertial measurement unit (IMU) and a photoplethysmography (PPG) unit. The IMU contains a 3-axis accelerometer of a sampling rate of 50 Hz and a resolution of 16 bits with LSBs of 0.061035 · 10 − 3 g (accelerometer) and 0.01526 dps (gyroscope). The PPG module is capable of measuring the heart rate and blood oxygenation at an 8-bit resolution.

Captured raw data are transmitted via Wi-Fi and can be accessed by using the free software development kit provided by MindRove^46^. Alternatively, they can be displayed, saved and transmitted via Lab Streaming Layer (LSL) by using the Visualizer on Desktop application.

#### mBrainTrain SMARTING

The mBrainTrain SMARTING (mBrainTrain, Belgrade, Serbia^47^) is also a mobile EEG system compatible with standard EEG caps with passive electrodes. Particularly, the use of EASYCAP products is recommended^48^, the official web page of the device^47^ features two separate layout. The device supports recording data up to 24 channels. The montage follows the electrode configuration of the applied EEG cap. In this research, the motor layout cap (EASYCAP GmbH, Herrsching, Germany^49^) was utilized with reference and DRL electrodes at FCz and AFz standard positions, respectively. The sensors are manufactured from sintered silver/silver chloride. The montage is depicted in Fig. 1b. The device along with the EEG cap is shown in Fig. 1c.

Samples are collected at a sampling rate of 500 Hz and a resolution of 24 bits and are transmitted via Bluetooth (version 2.1^48^). Motion tracking is enabled by the built-in 3-D gyroscope module. Data can be recorded or transmitted via LSL by using the SMARTING Streamer utility.

SMARTING is widely applied in research where a mobile EEG data acquisition device is needed. Examples include the measurement of event-related potentials related to musical note sequences^50^ and words pertaining to separate writing systems^51^, assessment of task load^52^ and interbrain synchrony^53^, and implementation of biofeedback/neurofeedback^54,55^ and synchronised EEG and eye tracking^56^ systems. SMARTING also has been utilised as a reference device that the performance of OpenBCI Cython + Daisy board was compared to^57^. The device is most commonly equipped with EASYCAP products ^50–54,56^; notable exceptions feature flexible EMG electrodes^55,58^ or cEEGrid that record EEG signal placed around the ear^56,57^.

### Subjects and data acquisition protocol

**Fig. 8:**
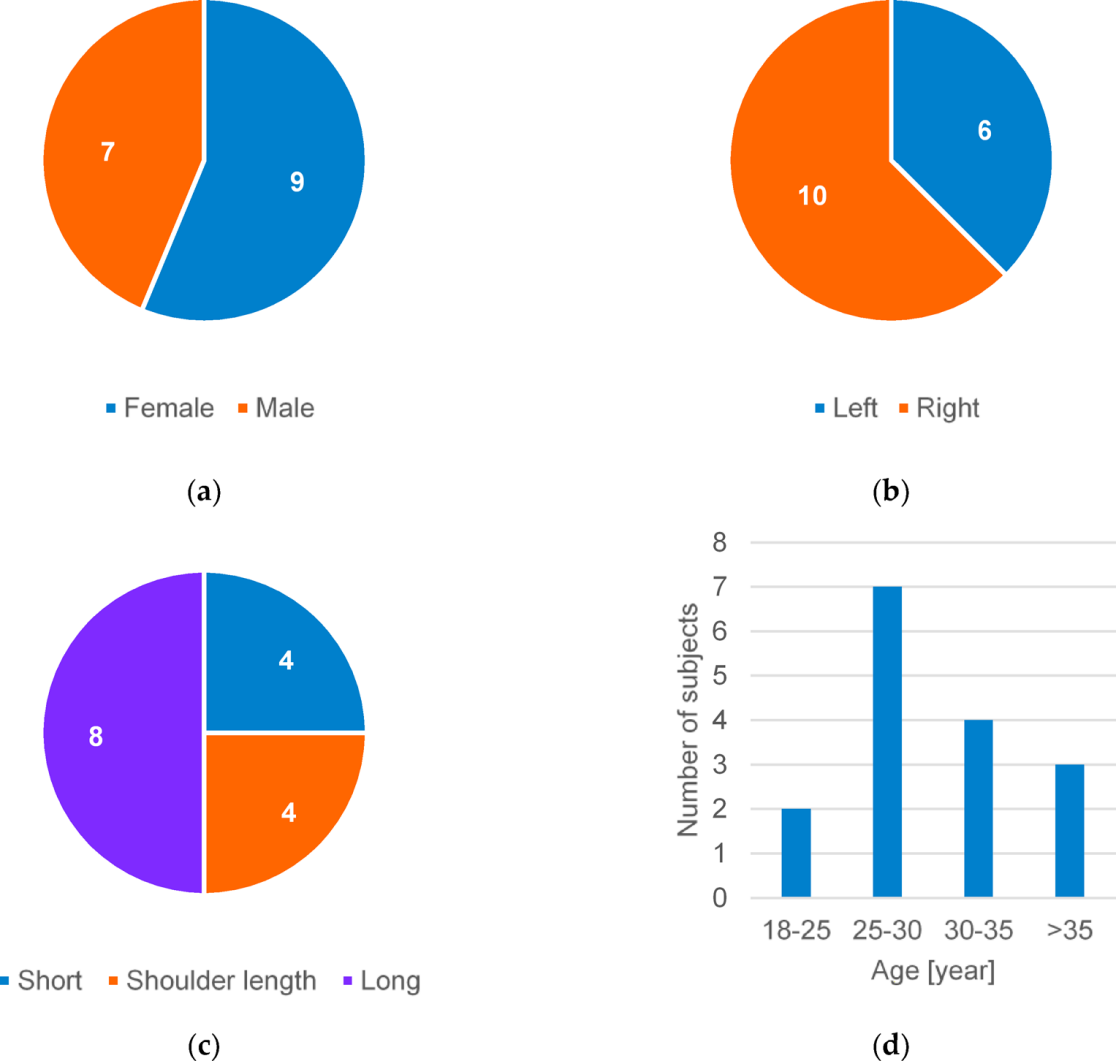
Basic characteristics of subject population. Distribution of subjects by (**a**) gender, (**b**) handedness, (**c**) hair length and (**d**) age.

16 subjects were recruited for the experiment by using convenience sampling. None of them had a history of neurological or visual processing disorder; all of them had normal or corrected-to-normal vision. 11 participants (68.75%) were research fellows and/or students working at the same institute as the first author. 56.25% of the subject was female, 62.5% right-handed and 75% had at least shoulder-length hair. The age of all participants was above 18 (between 21 and 54) years (30.875 ± 8.632); due to the prevalence of young researchers in the subject pool, 43.75% of the participants fell into the 25–30 age gap, making it the most prevalent one. Main characteristics of the subject pool are demonstrated in Fig. 8.

**Fig. 9:**
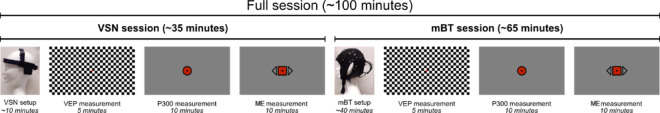
Schematic depiction of the experimental protocol. Measurements were organized into blocks according to the data acquisition instrument applied. MindRove vision was set up first, followed by mBrainTrain SMARTING (as the latter required electrolyte gel for proper working). In each block, the order of paradigms was the same (VEP–visually evoked potentials, P300–event-related potentials, ME–motor execution).

Upon arrival, subjects were instructed to read the informed consent form, and, in case of agreement, to complete it. Then, VSN was installed to the subjects’ head, with its electrodes wetted with tap water, followed by a complete set of measurements. Before each measurement, electrode impedances were lowered so they did not exceed 150 kΩ (the recommended limit given by MindRove^59^) by adjusting band tension and contact pressure, repositioning the electrodes and separating hair. Prior to each measurement, subjects were quickly introduced to the paradigm in question. After the first series of measurements, VSN was removed and mBT got installed by using the SUPER-VISC high-viscosity electrolyte gel (EASYCAP GmbH, Wörthsee, Germany). To keep setup time the shortest, only electrodes matching the montage of VSN (i.e. Fp1, Fp2, C3, Cz, C4, O1, O2, TP9 and TP10) were set this way. Impedances were set not to exceed 10 kΩ. Then the entire measurement set was repeated by using mBT. After finishing the second series of measurement, subjects were instructed to wash their hair and were rewarded with a chocolate bar or dried fruit snack (vegan option) for participation. During the full course of the session, subjects were free to pause or abort the measurements, but everyone completed the process. The structure of the protocol is illustrated in Fig. 9.

During preprocessing, mBT data were re-referenced to TP9 to match the electrode configuration of VSN.

### BCI paradigms

**Fig. 10:**
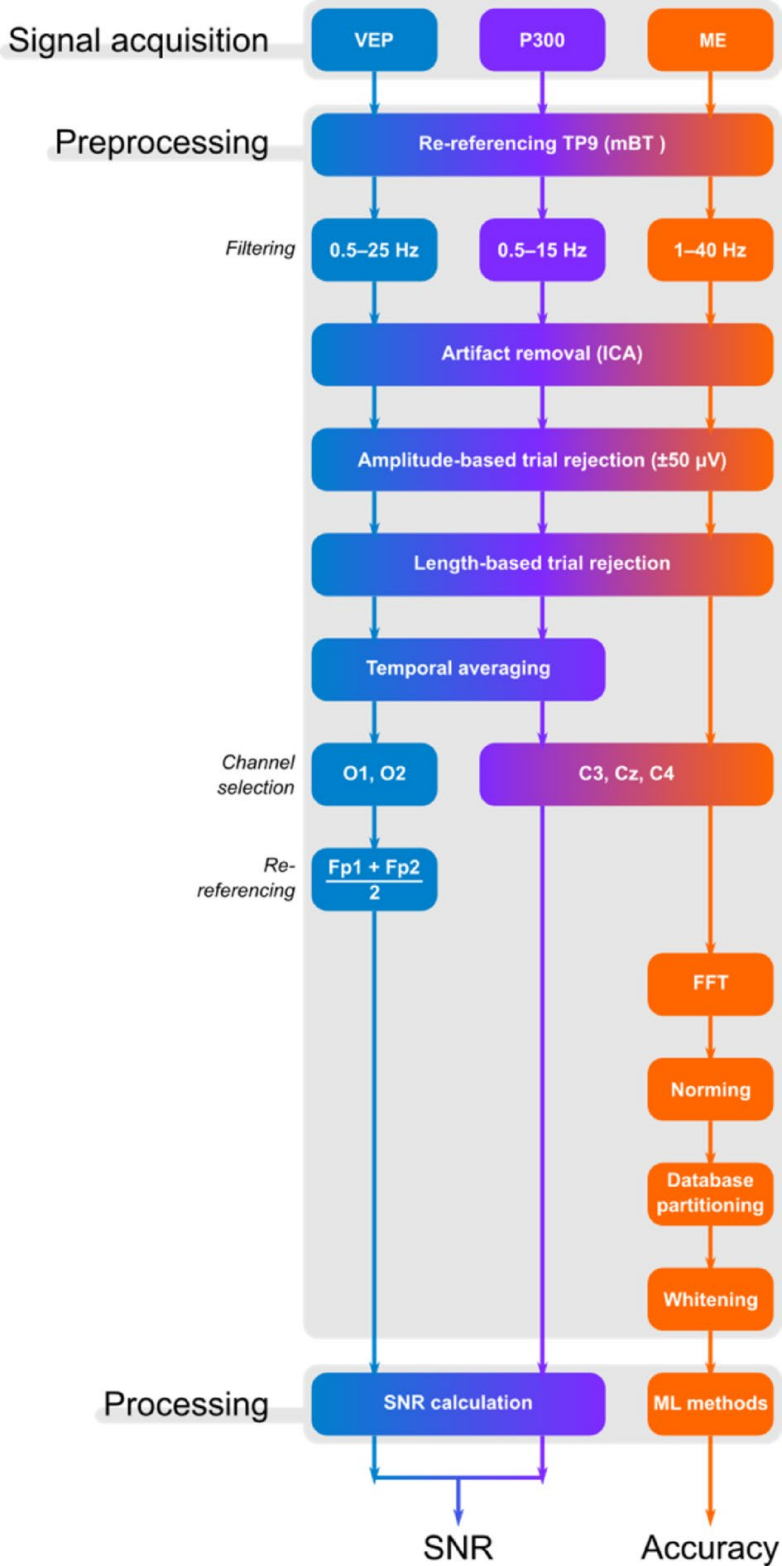
Data acquisition, preprocessing and processing steps for data recorded via one of the three paradigms. For a detailed description, see the text.

This section describes the data acquisition paradigms we applied during our investigation, along with the details on data preprocessing, i.e. the steps prior to data quality assessment. Our process is illustrated in Fig. 10.

Data were obtained via one of the visually evoked potentials (VEP), oddball (P300) or motor execution task (ME) paradigms. Then they underwent a few pre-processing steps that were the same for all data: task-specific filtering, artifact removal by using independent component analysis (ICA), the rejection of data exceeding a specific threshold (± 50 µV) possibly signifying electrode popping or other recording artifacts and the rejection of trials of insufficient length (due to communication error and packet loss). Data recorded by using mBT were re-referenced to TP9 prior to these steps. Then, data went through a few task-specific processing steps. In order to obtain the signal-to-noise ratio, VEP and P300 samples were averaged and the channels of interests were kept for further analysis. ME data were transformed into the frequency domain and after splitting the dataset into training, validation and test databases, whitened.

Particular steps of our process will be discussed in greater detail in the following subsections.

#### Visually evoked potentials

Visually evoked potentials (VEP) are electrophysiological potentials that can be elicited by using visual stimuli; they can be extracted from EEG activity recorded at the scalp^60^. Evoked potentials are reliable and mostly stable measure of nervous system function^61^, indicating the presence of neurological disorders such as multiple sclerosis or optic neuritis, even in absence of any symptom^61^. Due to their small amplitude, averaging of multiple instances is necessary^60^.

VEPs can be evoked by using different types of flickering visual stimuli such as flashing light, pattern onset and pattern reversal^60^, each yielding a characteristic waveform. If the flickering frequency is above 3.5 Hz, the wave becomes continuous (known as steady-state VEP, ssVEP), containing frequencies that correspond to stimulus frequency and its harmonics^62^. Below this frequency, VEPs are transient, containing only a few deflections^60^. A pattern reversal VEP consists of a wave of 20 µV amplitude, with two negative peaks at approximately 75 and 135 ms after stimulus presentation and a positive peak at approximately 100 ms. The working electrode, ground and reference are commonly placed at Oz, Cz and Fz^60^.

**Fig. 11:**
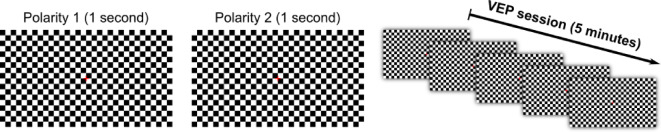
Schematic depiction of the visually evoked potential paradigm. During the 5-min course of the measurement, a chessboard pattern changed polarity in every second.

In brain–computer interfaces, mostly ssVEP paradigms get implemented, where checkerboards flickering with different frequencies correspond to different inputs, such as letters (see e.g.^63^ for a review). In this research, a pattern reversal transient VEP paradigm was implemented due to its results being the least variable in waveform and timing^60^. A 32 × 18 field (resolution: 1920 × 1080 px) checkerboard pattern changing polarity in every second (this corresponds to a 0.5 Hz frequency) was displayed on a screen of dimensions 34.3 × 19.3 cm the subjects sat 50 cm from. The subjects were instructed to fixate on a red cross placed at the center of the screen and abstain from any movement including blinking. The measurement lasted for 5 min corresponding to 300 pattern reversals. The paradigm is illustrated in Fig. 11.

Checkerboard stimuli were drawn in Inkscape. Data processing scripts were written in Python (version 3.9)^64^ by using Numpy (version 1.26.4)^65^, SciPy (version 1.13.0)^66^ and scikit-learn (version 1.5.0)^67^. Data were band-pass filtered between 0.5 and 25 Hz using second-order-section (SOS) filters formed by concatenating a second-order high-pass filter and a second-order low-pass filter. This frequency range preserves the transient VEP components (N75, P100, N145), which lie predominantly below 25 Hz, while reducing slow baseline fluctuations and high-frequency noise. Then blinking and eye movement artifacts were removed via independent component analysis (using the FastICA implementation of scikit-learn), using 7 components and a random state of 23. Components were removed upon manual inspection. ICA was followed by the rejection of trials (corresponding to reversals) exceeding a ± 50 µV threshold^60^. Trials of insufficient length (resulting from packet loss) got also rejected. Then, trials were averaged; channels corresponding to O1 and O2 and re-referenced to the average of Fp1 and Fp2 were used for subsequent analysis. For additional details, please refer to the supplementary material available at https://github.com/MelindaRacz/VSN_validation_supplementary/blob/main/Preprocessing/VEP.py.

#### Event-related potentials

According to Regan, event-related potentials (ERPs) are transient wave complexes elicited by a certain single (i.e. not repeating) stimulus or event^68^. P300 (or P3) is one of the most widely investigated ERPs; it is a parietocentral positivity that occurs when a subject detects an informative task-relevant stimulus^69^. P300 is also frequently utilized in brain-computer interfaces implementing mental typing^70–73^. Most commonly, rows and columns are highlighted in a 2-dimensional array of characters and the presence of a P300 wave signifies the letter (or group thereof) of interest. Due to the small amplitude of the potential (approximately 10 µV^69^), instances are averaged to extract the wave.

**Fig. 12:**
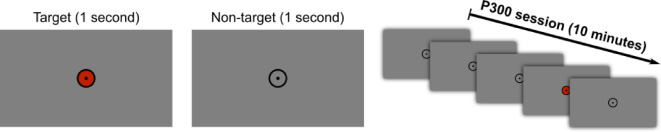
Schematic depiction of the event related potential paradigm. During the 10-min course of the measurement, 600 one-second-long stimuli were presented with a target probability of 10%. The start of each trial was indicated by a beep.

In this research, a visual oddball paradigm was implemented, with circles of red and grey fill as the target and non-target stimuli, respectively. Both features were placed at the center of a screen of dimensions 34.3 × 19.3 cm with grey background. At the start of each trial, a beep of 440 Hz lasting for 100 ms was presented. Individual trials lasted for 1 s and 600 trials was included in each round, thus measurements lasted for 10 min. Target probability was set to 10%, thus 60 target trials took place during one measurement. The subjects sat 50 cm from the screen. They were instructed to fixate on a black cross placed at the center of the screen and avoid any movement including blinking. Their task was to press the space key on the keyboard and to count target occurrences. The paradigm is illustrated in Fig. 12.

Stimuli were drawn in Inkscape. Data processing scripts were written in the same environment as our VEP processing software (using the same language and library versions). Data were band-pass filtered between 0.1 and 15 Hz using an SOS-based filter obtained by concatenating a second-order high-pass and a second-order low-pass filter. This low-frequency band was chosen to preserve the slow centro-parietal P300 component while attenuating baseline drift and higher-frequency contamination that does not contribute to ERP morphology. Then blinking and eye movement artifacts were removed via independent component analysis using 7 components and a random state of 23. Components were removed upon manual inspection. ICA was followed by the rejection of trials exceeding a ± 50 µV threshold^60^. Trials of insufficient length (resulting from packet loss) got also rejected. Then, trials were averaged separately by condition (i.e. target or non-target); channels corresponding to C3, Cz and C4 were used for subsequent analysis. For additional details, please refer to the supplementary material available at https://github.com/MelindaRacz/VSN_validation_supplementary/blob/main/Preprocessing/P300.py

#### Motor execution task

During the planning and the execution of a specific movement, event-related synchronization and desynchronization (ERS/ERD) patterns emerge^74,75^. This happens regardless of the ability to perform the physical activity in question. This provides the theoretical basis for motor imagery (MI)-based BCIs. Solutions commonly utilize hand^76^, foot^77,78^ and tongue^79^ movement, exploiting the good separability of the corresponding cortical patterns^75^.

**Fig. 13:**
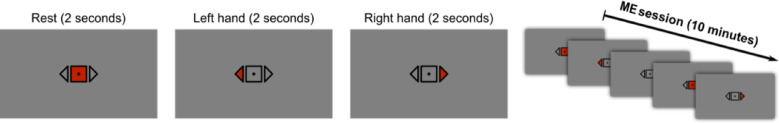
Schematic depiction of the motor execution paradigm. During the 10-min course of the measurement, 300 two-second-long stimuli were presented. The start of each trial was indicated by a beep.

In this research, the implemented a motor task paradigm involved the movement of the two hands. Three features were placed against the grey background of a screen of dimensions 34.3 × 19.3 cm: a rectangle at the center of screen and two triangles at its left and right sides. During every trial, exactly one of the features was filled with red, corresponding to the task in question, the others with grey. Rest was signified by highlighting the rectangle, while moving the left and the right hand by highlighting the triangle facing left or right, respectively. Individual trials lasted for 2 s; rest, left-hand and right-hand movement cues were presented 120, 90 and 90 times, respectively, thus measurements lasted for 10 min. At the start of each trial, a beep of 440 Hz and lasting for 100 ms was presented. The subjects sat 50 cm from the screen. They were asked to fixate on a black cross placed at the center of the screen and abstain from any movement including blinking. Their task was to sit relaxed, make a fist with their left or right hand then release it, corresponding to the prompt. Real movements were performed instead of their imaginary counterparts due to their easier execution. The paradigm is illustrated in Fig. 13.

Stimuli were drawn in Inkscape. Data processing scripts were written in the same environment as our VEP processing software (using the same language and library versions). Data were filtered between 1 and 40 Hz Hz by using 1 s-order low- and 1 s-order high-pass sos filter that were concatenated. This band was selected to retain the sensorimotor mu (8–13 Hz) and beta (13–30 Hz) rhythms, which are known to carry the most discriminative information for motor execution tasks, while attenuating slow drifts (< 1 Hz) and high-frequency muscle or environmental noise (> 30–40 Hz). Following frequency-domain transformation, only the 8–30 Hz band was retained for feature extraction to focus specifically on these task-relevant oscillatory components. Then blinking and eye movement artifacts were removed via independent component analysis using 7 components and a random state of 23. Components were removed upon manual inspection. ICA was followed by the rejection of trials exceeding a ± 50 µV threshold^60^. Trials of insufficient length (resulting from packet loss) got also rejected. Then, each 2-s trial samples were transformed into frequency domain (using the SciPy implementation of Fast Fourier Transform), as part of frequency-domain feature extraction. Components between 8 and 30 Hz (according to Afrakhteh and Mosavi^80^ and channels corresponding to C3, Cz and C4 were kept for subsequent analysis. Samples were normed by using Eq. (1), where $\acute{x}_{{i_{{ch}} }}$ and ${x}_{{i}_{ch}}$ stand for the sample after and before norming, respectively, in a set of samples corresponding to left- or right-hand movement, or rest. In other words, samples belonging to separate movement types were normed together.

1 $$\acute{x}_{{i_{{ch}} }} = ~\frac{{x_{{i_{{ch}} }} - \min x_{{i_{{ch}} }} }}{{\max x_{{i_{{ch}} }} - \min x_{{i_{{ch}} }} }}$$

After norming, data were shuffled and then partitioned into training and test data by using an 80/20% split. After this step, training and test data were whitened separately according to Eq. (2), where $\acute{x}$ and $x$ stand for data after and before whitening, respectively, $\mu$ for the mean and $\sigma$ for the standard deviation of x.

2 $$\acute{x}= \frac{{x - \mu }}{\sigma }$$

For additional details, please refer to the supplementary material available at https://github.com/MelindaRacz/VSN_validation_supplementary/blob/main/Preprocessing/MI.py

### Machine learning methods

#### Support vector machines

Support vector machines^81^ are one of the most popular machine learning methods used in motor imagery BCIs. The algorithm aims the separation of data points supposedly belonging to two classes, by a maximum margin, using a hyperplane. The method can be generalized to data points not linearly separable by using the kernel trick^82^, and to multiple classes by utilizing multiple (one-versus-one or one-versus-rest) classifiers^83^.

In this research, the scikit-learn implementation of SVM, more specifically, nu-SVC was applied. Nu-SVC prescribes a fraction of margin errors (i.e. nu) that is permitted to occur^84^. Nu was chosen 0.1, the kernel ‘radial basis function’, the class weight ‘balanced’, the decision function ‘one-versus-one’ and the tolerance 0.001. As the method does not exploit spatial dependences, samples were flattened prior to feeding to the algorithm.

#### Random forest

Decision trees classify data based on decisions regarding the most salient features of a given dataset (i.e. splitting it into parts by using questions related to certain parameters)^85^. Random forests^86^ use an ensemble of decision trees to prevent overfitting. Samples will be labelled according to the majority vote of the individual trees.

In this research, we used the scikit-learn implementation of the random forest algorithm (RandomForestClassifier). We chose the number of estimators 100, the decision criterion ‘entropy’. As the method does not exploit spatial dependences, samples were flattened prior feeding to the algorithm.

#### Convolutional neural networks

Convolutional neural networks (CNNs)^87^ are a subtype of artificial neural networks that extract local dependencies of their input (e.g. edges in an image) that can be combined into complex structures (e.g. representation of image classes such as human faces) if the network contains a sufficiently large number of layers^88^. In the recent years, CNNs are getting more popular in relation to EEG signal processing, thus MI-BCIs, as well^89^.

**Fig. 14:**
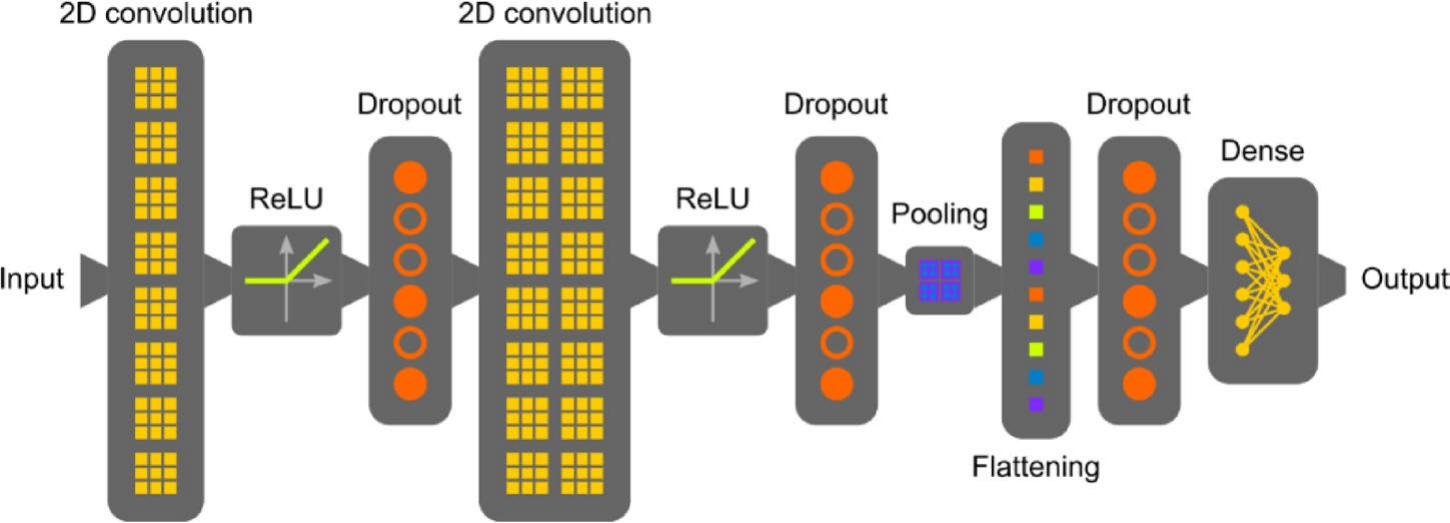
Architecture of the CNN. The first convolutional layer contains eight 3 × 3 filters, the second 16. Both layers utilize ReLU activation and are followed by dropout layers (with 50% dropout probability). After the second layer, 2 × 2 pooling, flattening and a third dropout is applied. Samples get finally classified by a dense layer with 3 output neurons.

In this research, a three-layered convolutional network was implemented in Keras^90^ (version 3. 3. 3). The first two layers were convolutional, using 3 × 3 kernels, ReLU activation, zero padding (keeping the dimensions the same between layers) and a dropout of 0.5. The number of filters in the first and second layers were 8 and 16, respectively. After the second layer, a 2D max pooling of 2 × 2 was implemented, followed by flattening and another dropout of 0.5. The final layer was dense, differentiating between the 3 classes (corresponding to rest, right and left-hand movement). Network architecture is depicted in Fig. 14.

During training, categorical crossentropy was applied as loss, and Adam as optimizer. We implemented early stopping, monitoring the accuracy on the validation dataset. The maximum number of epochs was set to 2500; a patience of 1000 was also introduced, starting from epoch 25. After the training, the classifier was rolled back to the best iteration. The percentage of the test sample pool was the same as for SVM and RF (i.e. 20%), whereas the validation dataset was split off the training database, consisting of 25% of the available samples. This resulted in a 60/20/20% training/validation/test split.

### Evaluation metrics

#### Signal-to-noise ratio

**Fig. 15:**
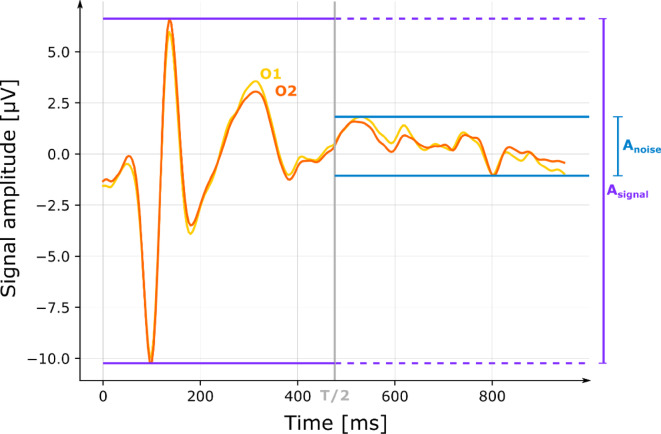
Signal-to-noise ratio computation for VEP. The first half of the 1-s-long time window (i.e. before T/2) containing the average of all samples was supposed to include the majority of the VEP wave. The peak-to-peak amplitude of the two channels of interest (O1 and O2) was taken as the signal amplitude (A_signal_). Signal amplitude in the second half of the time window was supposed to contain only noise, thus its peak-to-peak value was taken as the noise amplitude (A_noise_). This figure was prepared from data acquired from a single subject, using VSN.

The quality of VEP and P300 measurements was assessed by using the signal-to-noise ratio (SNR) that was calculated differently for the two potentials. For VEP, we did not define target and non-target conditions. Therefore, different parts of the one-second-long time window (i.e. corresponding to the presentation of the stimulus of one polarity) were used for computing signal and noise amplitude. Prior to the calculation of SNR, all samples were averaged. Since the reference electrode is commonly located at frontal sites at VEP measurements^60^, channels of interest (O1 and O2) were re-referenced to the average of frontal channels (Fp1 and Fp2). Then, peak-to-peak values in the first and second half of the time window were computed, with the first half supposedly containing the waveform of interest, and the second half only noise, assuming that the decay of the VEP wave has been completed. These considerations are illustrated in Fig. 15.

SNR was then computed by using Eq. (3), where $SN{R}_{ch}$ stands for the SNR on an individual channel (O1 or O2), ${A}_{signa{l}_{ch}}$ and ${A}_{nois{e}_{ch}}$ for the amplitude of the signal and noise in the same channel, respectively, and $SN{{R}_{dB}}_{ch}$ for SNR in decibels.

3 $$SN{R}_{ch}=\frac{{A}_{signa{l}_{ch}}}{{A}_{nois{e}_{ch}}} \; SN{{R}_{dB}}_{ch}= 20\cdot {\mathrm{l}\mathrm{o}\mathrm{g}}_{10} SN{R}_{ch}$$

**Fig. 16:**
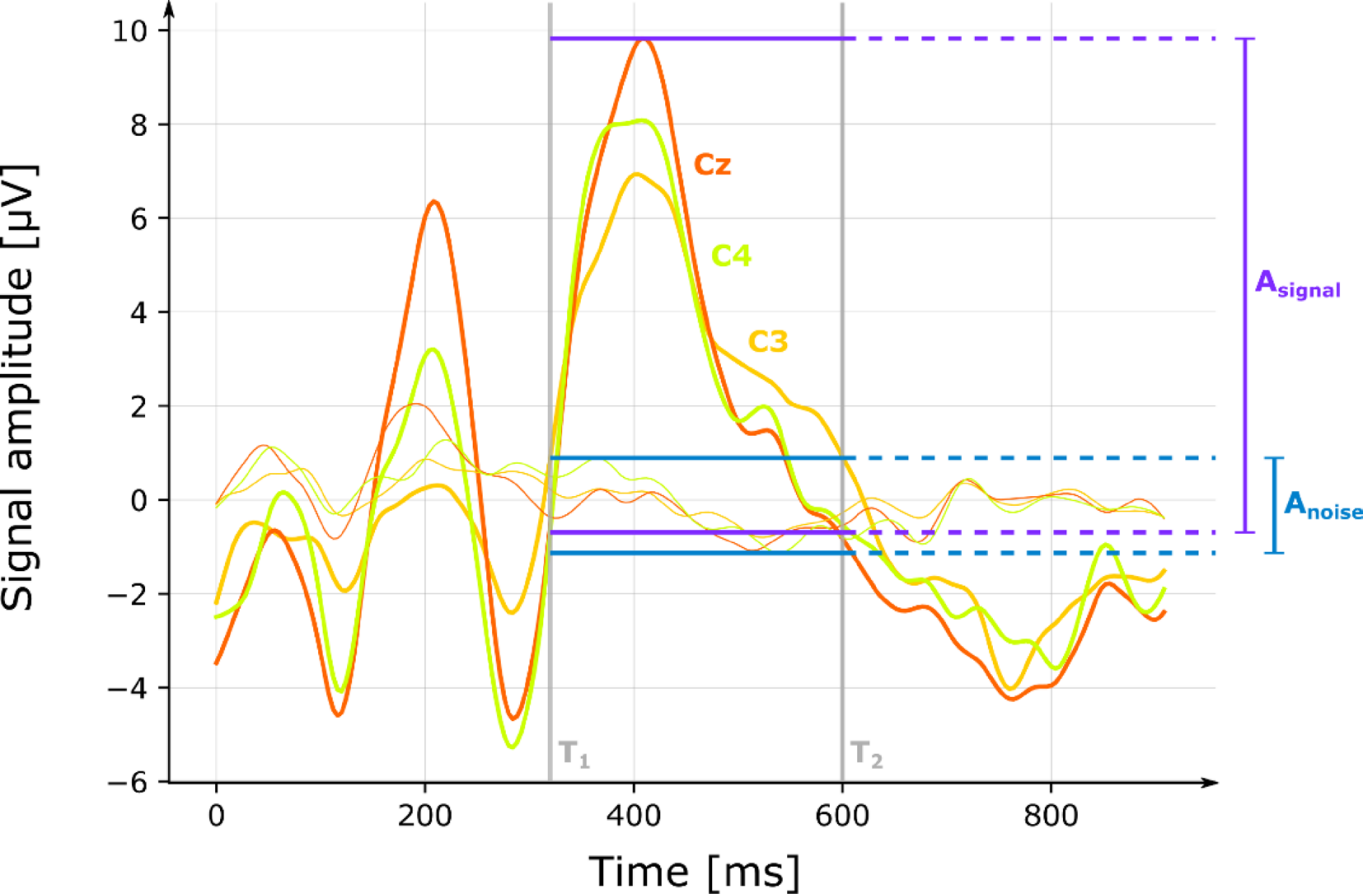
Signal-to-noise ratio computation for P300. The part of the 1-s-long time window containing the average of all target samples, that supposedly includes the P300 wave (i.e. the range between T1 and T2) was determined manually for each subject. Peak-to-peak amplitude of the three channels of interest (C3, Cz and C4) given the average of target samples (thick traces) was taken as the signal amplitude (A_signal_). Signal amplitude in the same segment given the average of non-target samples (fine traces) was supposed to contain only noise, thus its peak-to-peak value was taken as the noise amplitude (A_noise_). This figure was prepared from data acquired from a single subject, using VSN.

Unlike for VEP, we had two different conditions for P300 measurements, thus, target and non-target samples could be separated better. Accordingly, target and non-target samples were averaged separately. Then, P300 waves were identified manually for each subject with their amplitude measured (signal). Peak-to-peak values within the same segment were also computed for non-target average (noise). Sampling is demonstrated in Fig. 16. SNR was computed by using Eq. (3) for the channels of interest (C3, Cz and C4).

#### Accuracy

The accuracy of a classifier is defined as the percentage of correctly classified samples. It is expressed in Eq. (4), where $T{P}_{i}$ stands for the number of correctly classified (true positive) and $F{N}_{i}$ for the misclassified (false negative) samples in a given class and $N$ for the number of classes.

4 $$A=\frac{\sum_{i=1}^{N}T{P}_{i}}{\sum_{i=1}^{N}\left(T{P}_{i}+F{N}_{i}\right)}\mathrm{o}\mathrm{r}\,A=\frac{\sum_{i=1}^{N}T{P}_{i}}{\sum_{i=1}^{N}\left(T{P}_{i}+F{N}_{i}\right)}\cdot 100 \%$$

For each classifier and device and subject, 5 training-test runs were performed and their average accuracy was taken.

### Statistical analysis

We assessed the differences between the following variables given the two devices: peak-to-peak signal (VEP or P300) values, peak-to-peak noise values, SNR and SNRdB. For the assessment of these differences, we applied the measurement equivalence assessment developed by Silveira et al.^91^. Tests were applied to values averaged across channels, in a bootstrapped fashion (given 2000 re-samplings).

When it comes to the assessment of equivalence of two measurement methods, one can speak of strict (based on statistical inference) and flexible or practical equivalence (based on clinical importance)^92^. Contrary to the traditional approach proposed by Bland and Altman^93^, that relies on visual inspection to assess flexible/practical equivalence (where the decision can be subjective in nature), the procedure adopted here (developed by Silveira and colleagues) provides clear null hypotheses involving three main steps to test strict equivalence of the devices.

1. *Accuracy* refers to the equivalence of means (i.e. location shift or systematic bias). Instead of a paired t-test, a more powerful method was applied (described by Hedberg and Ayers^94^), where differences between measurements from the two devices (${y}_{i}-{x}_{i}$) were regressed on the centered value of the reference measurement (${x}_{i}- \overline{x }$), as expressed in Eq. (5).
  5 $$y_{i} - x_{i} = \alpha + \beta \left( {x_{i} - \overline{x}} \right) + \varepsilon_{i}$$
  Since intercept is the indicator of bias, $\alpha =0$ means an absence of bias. H0 (no bias) is rejected if the point (0,0) is outside the 95% confidence interval.
2. *Precision* refers to equal variability of the measurement errors given the two devices. To test the H0 of equal precision, a linear regression was applied^95^ as expressed in Eq. (6).
  6 $$y_{i} - x_{i} = \alpha + \beta \left( {x_{i} + y_{i } /2} \right) + \varepsilon_{i}$$
  A slope of zero ($\beta =0$) indicates equality, consequently the H0 of equal precisions is rejected if a horizontal line cannot be fitted into the 95% confidence band.
3. *Reliability* in terms of bisector line agreement. Deming regression^96^ was performed to test whether the two devices are interchangeable (i.e. Y = X). Reliability is expressed in Eq. (7).
  7 $$y_{i} - x_{i} = \alpha + \left( {\beta - 1} \right)x_{i} + \delta_{i} - \beta \varepsilon_{i}$$
  Here ${\delta}_{i}$ and ${\varepsilon}_{i}$ are the error terms. In contrast to ordinary least squares, the Deming regression assumes that both measurement techniques have measurement errors and take these into account. The H0 of agreement ($\alpha =0$, $\beta =1$) is rejected if the bisector line cannot be fit inside the 95% confidence band.

For the assessment of accuracy differences given the various machine learning classifiers, we fitted a linear mixed-effects model (LMM) with device (VSN and mBT) and algorithm (SVM, RF and CNN) type as fixed effects and subject as random effect. We assumed a fixed-slope model with random intercepts. Based on visual inspection of the variability of accuracy with respect to subjects, we tested two LMMs assuming same and different variance for each subject, respectively. In addition to the additive models mentioned above, we tested an LMM that considered interaction between device and algorithm type (with different variance for each subject).

Performance of models was compared by using the likelihood ratio test^97,98^. Prior to the test, they were re-fitted by using a maximum likelihood method. Apart from this, LMMs applied the restricted maximum likelihood method.

All statistical analysis scripts were implemented in R^99^. For linear mixed models, the nlme package (lme function) was used^100^. To assess measurement equivalence, we used the eirasagree package^101^.

## Conclusions

These results support the use of the paste-less electrode-based EEG headset for acquiring VEP, P300, and motor-execution-related EEG signals in BCI-relevant settings. While VEP and P300 were assessed primarily through signal-quality measures, the motor task was additionally evaluated by classifier performance, which did not differ significantly between devices in the selected statistical model. Nevertheless, further work is needed to establish end-to-end BCI performance across all paradigms. This assumption was corroborated by using MindRove vision that exhibited signal levels comparable to that of mBrainTrain SMARTING and existing literature. We hope this article contributes to research targeting the accessibility of brain–computer interfaces and other devices aiming at preserving and improving the well-being of a wide range of users in the future.

## Data Availability

Measurement data, preprocessing scripts, additional figures and details on statistical analysis are provided as supplementary material to this article, made accessible at (https://github.com/MelindaRacz/VSN_validation_supplementary). Other details are provided upon request addressed to the corresponding author.
